# Therapeutic Cancer Vaccines in Prostate Cancer: The Quest for Intermediate Markers of Response

**DOI:** 10.3390/cancers4041229

**Published:** 2012-11-21

**Authors:** Joseph W. Kim, Marijo Bilusic, Christopher J. Heery, Ravi A. Madan

**Affiliations:** 1 Laboratory of Tumor Immunology and Biology, Center for Cancer Research, National Cancer Institute, National Institutes of Health, Bethesda, MD 20892, USA; 2 Medical Oncology Branch, Center for Cancer Research, National Cancer Institute, National Institutes of Health, Bethesda, MD 20892, USA

**Keywords:** surrogate marker, immune assay, tumor-infiltrating lymphocytes, tumor growth rate

## Abstract

Despite recent advances in cancer immunotherapy, no prospectively validated intermediate biomarkers exist to predict response. These biomarkers are highly desirable given modern immunotherapy’s paradoxical pattern of clinical benefit; that is, improvement in overall survival without short-term change in progression. Immunotherapy clinical trials have evaluated biomarkers that may correlate with clinical outcomes. Many of them are performed on peripheral blood to evaluate the systemic response, such as tumor-targeted humoral and cellular immunity, and cytokine responses. Accumulating evidence suggests that immune infiltrates in tumors may suggest evidence for the therapy’s mechanism of action, and have greater potential for providing prognostic and predictive information. In addition, a non-immunologic biomarker, such as tumor growth kinetics, may explain this paradoxical pattern of clinical benefit, and predict survival in patients treated with an immunotherapy. Prospective assessment and validation of these and other intermediate markers would be required to better understand their potential clinical role.

## 1. Introduction

After decades of preclinical research and failure in early clinical trials with rudimentary immune-stimulating treatments, modern immunotherapies have emerged that demonstrated therapeutic efficacy. Phase III trials of sipuleucel-T and ipilimumab have extended survival in patients with late-stage prostate cancer and melanoma, respectively [1,2]. In spite of these successes, these agents have posed new and difficult quandaries for clinicians. Unlike the standard cytotoxic agents practitioners are familiar with, modern immunotherapies appear to work in a different and more delayed fashion. In what appears to be a paradox, neither sipuleucel-T nor ipilimumab has shown changes in short-term disease progression, but each has demonstrated long-term improvements in survival.

Sipuleucel-T, the first FDA-approved vaccine for the treatment of prostate cancer, demonstrated a 4.1-month improvement in overall survival relative to placebo in patients with metastatic castration-resistant prostate cancer [1] (consistent with contemporary emerging therapeutics). This was the second trial of sipuleucel-T to demonstrate this apparent delayed therapeutic effect. Similarly, ipilimumab improved survival in metastatic melanoma by a clinically meaningful 3.5 months. Both these agents however, demonstrated no improvement in short-term time to progression at 3 months compared to placebo [2]. In a phase II trial, PSA-TRICOM, another prostate cancer vaccine, demonstrated a similar pattern of improved survival without short-term changes in progression [3]. These findings are inconsistent with standard cytotoxic therapies, which generally demonstrate short-term improvement in disease progression, with the potential for improved survival. Nonetheless, the findings from trials with these three agents could represent the hallmark of modern immunotherapeutics. These clinical outcomes could be manifestations of how these therapies are different from standard cytotoxic therapies that do not require time to initiate an immune response [4].

Although these new therapies may work differently, clinicians must still be able to evaluate their efficacy in the near term to determine the most appropriate treatment for their patients. Although overall survival remains the gold standard, it is a distal endpoint. Therefore, the quest for intermediate biomarkers of immunologic response has never been more important. Now that immunotherapies have demonstrated clinical efficacy, clinicians need to be armed with methods of assessment that can identify responders and nonresponders to treatment. Many assessment techniques have been tested over the years, demonstrating their various strengths and shortcomings. It is now imperative that we understand these assessment tools in the context of clinical practice, to help us determine which intermediate biomarkers have the greatest potential as surrogates for clinical response and overall survival. Therefore, the discovery for potential intermediate biomarkers should begin with examination of overall survival data from randomized clinical trials. However, given that only a few a therapeutic cancer vaccine trials have such data, the task is difficult.

## 2. Immunological Assays

Many clinical trials of a therapeutic cancer vaccine have utilized immunological assays to assess immune response and to correlate the findings with clinical outcome. Table 1 provides an overview of selected immune assays.

**Table 1:** Overview of selected immune assays.

	Description	Limitations
Multimer Assay	- Tests the antigen recognition of T cell receptor. - Able to detect low-population antigen specific T cells. - Provides quantification of antigen specific T cells	- Does not provide functional information
T cell proliferation assay	- Tests ability of T cell to proliferate in response to an antigen of interest - Relatively easy to perform - Can be analyzed via flow cytometry	- Identity and function of the proliferating cells are unknown - May detect bystander activation
Cytotoxic T Lymphocyte Assays		
*^51^Chromium release assay (CRA)*	- Tests cytotoxicity of T lymphocytes - Does not provide information of target cell death at single-cell level	- Handling of radioactive material - Low sensitivity
*Caspase 3 Assay*	- Detects CTL-induced apoptosis	- Not suited for caspase-independent pathway of target cell killing.
Cytokine Production Assays		
*ELISPOT*	Measures antigen-specific T-cell activation through gamma-interferon production	- Significant variability among institution - Not useful with whole tumor cell vaccines or non-specific immunotherapy
*Intracellular Cytokine Staining*	Flow cytometry based assay to provide information on functional role of CTL using fluorescent antibodies	- Nonspecific background staining
*ELISA*	Accurate and sensitive detection of the cytokine	-Performance is largely dependent on antibody quality, kit manufacturer, as well as operator skills and experience
*RT-PCR*	Evaluate cytokine production measuring mRNA in single cell	- Extreme sensitivity (measuring biologically insignificant, transcriptional “noise”) - Necessitates destruction of the T cell
Regulatory T cells	- Major role in suppressing other immune cells.	- FoxP3 is most accepted marker. May not detect presence of small population of FoxP3^−^ Tregs
Delayed Type Hypersensitivity	- Tests memory T cell immunity - Relatively easy to perform	- Lacks antigen-specificity - May require skin biopsies and additional immune analyses to study the phenotypes of the infiltrating immune cells.
Humoral Response	- Numerous methods of detection of serum antibodies	- Needs further evaluation
Tumor infiltrate lymphcoytes	- May provide direct evidence of anti-tumor immune response	- May not have sufficient amount of tissue for immune analysis

### 2.1. Delayed-Type Hypersensitivity

One of the earliest tests of immune response was delayed-type hypersensitivity (DTH), also known as type 4 hypersensitivity [5,6]. DTH occurs as a memory response to an antigen that the immune system has previously been exposed to. Following the intradermal injection of antigens in a soluble protein form, local dendritic cells (DCs) process and present the antigen in the context of MHC class II molecules. Antigen-specific CD4^+^ cells are then activated by interaction with antigen-presenting cells (APCs). The activated CD4^+^ T cells mediate immune response by releasing cytokines, resulting in increased vascular permeability and recruitment of monocytes and inflammatory cells to the injection site. The diameter of the resultant induration or erythema is measured 48 to 72 hours following the injection.

DTH has been used in many clinical studies to assess the immune response induced by a therapeutic cancer vaccine [7]. It is relatively easy to perform, and correlation with clinical outcomes has been observed in some small studies [8,9]. One drawback, however, is that DTH can be a non-antigen-specific response. In a study of a peptide-loaded DC vaccine in melanoma patients, DTH response did not correlate with antigen loading [10]. Conversely, in a study of a carcinoembryonic antigen-loaded DC vaccine, despite a negative DTH response, biopsy of the injection site showed T-cell infiltrates [11]. Furthermore, the DTH assay does not provide information about the identity or function of infiltrating T lymphocytes. Skin biopsies and additional immune analyses are required to determine the phenotype of these cells [12].

In a phase I trial, 12 patients with hormone- and chemotherapy-refractory prostate cancer were treated every 2 weeks for a total of four injections with a DC vaccine targeting prostate stem cell antigen (PSCA) and prostate-specific antigen (PSA) [9]. Five patients developed a positive DTH response after the fourth vaccination. With a median follow-up of 13.4 months, DTH positivity was associated with improved survival (*p* = 0.003).

In another study, 50 patients with stage III or IV melanoma were vaccinated with autologous DCs pulsed with an allogeneic melanoma cell lysate. A significant improvement in median overall survival was seen in DTH^-^positive patients *vs*. DTH-negative patients (33 months *vs*. 11 months, *p* = 0.0014) [13]. All treated patients with stage III disease were DTH^-^positive and remained alive and tumor-free for a median follow-up of 48 months. Furthermore, DTH^-^positive patients showed a reduction in the proportion of CD4^+^ TGF-β^+^ regulatory T cells (Tregs) compared to DTH-negative patients (1.54% *vs*. 5.78%, *p* < 0.0001).

### 2.2. Multimer Assays

Multimer assay is a flow cytometry-based analysis to detect antigen-specific T cells by employing multimers of specific antigen peptide-MHC complexes. These multimers, commonly in the form of tetramers of biotinylated peptide-MHC complexes, are synthesized with known antigen peptides. After staining with fluorescence-labeled multimers, the T cells are analyzed by flow cytometry to detect T cells that stain positively with the multimer complex [14]. T-cell receptors usually have a low affinity for their cognate peptide-MHC complexes, and the duration of interaction is only a few seconds [15]. Studies have shown that interaction of the variable chain of T-cell receptor (TCR) is more avid with oligomers than it is with monomers of peptide-MHC complex [16,17]. Furthermore, evidence suggests that oligomerization of peptide-MHC complexes plays a role in TCR recognition and activation [17,18]. Therefore, by employing multimers of peptide-MHC complexes, this technology allows the detection of antigen-specific T cells with higher sensitivity, and allows detection of relatively low-population T cells that are specific to an antigen of interest [14,19]. Flow cytometry analysis also quantifies multimer-positive cells [14]. This assay in and of itself does not provide functional information on multimer^+^ T cells.

In another clinical trial in HLA-A2 positive patients with high-risk castration-sensitive prostate cancer who were vaccinated with a PSA peptide, there was a significant inverse correlation between changes in serum PSA levels and differences in average tetramer measurements at baseline and at week 26 (*p* = 0.02) [20]. It is of note that although commonly used in clinical practice, the use of biochemical endpoints, such as PSAs, is not recommended for use as primary indicators of response to therapy [21].

In a phase I trial of a plasmid-based vaccine targeting preferentially expressed antigen in melanoma (PRAME) and prostate-specific membrane antigen (PSMA) in patients with advanced solid tumors, immune monitoring consisted of tetramer assay and enzyme-linked immunosorbent spot (ELISPOT) [22]. A majority of evaluable patients (15/24; 63%) showed *de novo* induction or increased frequency of PRAME-specific and PSMA-specific T cells at one or more time points during treatment. Furthermore, there was an association between the induction and persistence of antigen-specific T cells in blood above baseline levels and disease control, defined as stable disease by RECIST criteria, for ≥6 months.

In a clinical trial with 41 HLA-A2 positive patients with advanced melanoma who were vaccinated with a high-dose polyepitope vaccine, the proportion of tetramer responders in the high-dose group was significantly greater than in the low-dose group. In addition, melan-A tetramer-positive immunity was associated with increased time to progression and survival compared with nonresponders [23].

### 2.3. T-Cell Proliferation Assays

Another way to measure immune response is to assess the proliferation potential of T cells to a given antigen. Antigen-specific T-cell proliferation assay is a flow cytometry-based analysis of the functional capacity of CD4^+^ or CD8^+^ T lymphocytes to respond to an antigen of interest [24]. T-cell proliferation assays measure the ability of T cells to proliferate following stimulation with a given antigen for a certain period of time (e.g., 5 days) in the presence of a radio-labeled nucleotide such as bromodeoxyuridine (BrdU) or ^3^H thymidine. Incorporation of radio-labeled nucleotide in T-cell populations is analyzed by liquid scintillation (betaplate) counter. The degree of antigen-specific clonal T-cell expansion is commonly expressed as a stimulation index of the ratio of radio-labeled nucleotide incorporation by cells incubated with an antigen, compared with media control [25].

A major limitation is that this assay alone does not provide information about the identity and function of these proliferating cells. In other words, this assay does not differentiate between subsets of these immune cells. Some investigators have used flow cytometry analysis to address this limitation. By staining cells with fluorescence-labeled antibodies appropriate for a subset of T cells (e.g*.*, anti-CD4, anti-CD8) and anti-BrdU and analyzing them via flow cytometry, they have differentiated subsets of T cells (BrdU-positive) that proliferate in response to antigen stimulation [26,27].

In a randomized, placebo-controlled trial involving 512 patients with metastatic castration-resistant prostate cancer, men who received sipuleucel-T showed a statistically significant improvement in overall survival of 25.8 months *vs*. 21.7 months for placebo (HR of mortality 0.78, *p* = 0.03) [1]. At week 6, T-cell proliferation responses to immunizing antigen PA2024 were observed in 46/63 patients (73.0%) in the sipuleucel-T group and 4/33 (12.1%) in the placebo group. Proliferation responses to prostatic acid phosphatase (PAP) were observed in 15/55 patients (27.3%) *vs*. 2/25 (8.0%) in the placebo group. However, T-cell proliferation response did not significantly correlate with overall survival [28].

In a phase I study of a DNA vaccine encoding PAP in 22 patients with castration-sensitive prostate cancer, 41% of patients developed PAP-specific CD4^+^ and/or CD8^+^ T-cell proliferation, and 14% developed PAP-specific IFN-γ-secreting CD8^+^ T cells by ELISPOT assay [27]. There was a trend toward improved PSA doubling time (PSADT), from a median of 6.5 months pretreatment to 8.5 months on treatment (*p* = 0.033) and 9.3 months in the 1-year post-treatment period (*p* = 0.054). Although baseline PSADT is a prognostic factor in untreated patients, change in PSADT is not an established prognostic tool. There were no major PSA responses (>50% decline) in this study, and no correlation was reported between immune response and clinical outcome.

### 2.4. Cytotoxic T Lymphocyte Assays

Cytotoxic T lymphocyte (CTL) assay is another method of assessing the cytotoxicity of CD8^+^ T lymphocytes. CTLs can play a crucial role in antitumor immunity by eliminating host cells undergoing malignant transformation. Traditionally, ^51^Cr release assay (CRA) has been used to quantify antigen-specific CTL activity [29]. Target cells expressing an antigen of interest are tagged with radio-labeled chromium. Effector cells collected from patients are then incubated with the target cells, allowing for lytic activity and release of ^51^Cr. The supernatant recovered from the assay is then analyzed to measure the level of ^51^Cr and to calculate the percentage of lytic activity. Although it is reproducible and relatively easy to perform, drawbacks of CRA include the use of radioactive material and the assay’s relatively low sensitivity. Furthermore, because it measures the “lytic unit,” it does not quantify target-cell death at the single-cell level [30].

In a study with a PSA peptide-based vaccine in 28 patients with locally advanced or metastatic prostate cancer, patients were vaccinated either by intradermal injection of PSA-peptide and GM-CSF or by intravenous administration of autologous DCs pulsed with PSA-peptide [12]. Fifty percent of patients developed DTH responses to PSA-peptide. Skin biopsies from seven DTH-positive patients were available for testing. Purified CD4^−^CD8^+^ T cells isolated from four of these biopsies demonstrated specific cytolytic activity per CRA [12]. In a long-term follow-up report, 13 patients had stable or declining serum levels of PSA one year post-vaccination. There was a trend toward greater overall survival in men who developed specific T-cell immunity [20].

An alternative to CRA is a flow cytometry-based CTL assay using the cleavage form of caspase 3 in target cells as a read-out. This assay is based on the understanding that the cytotoxicity of CTLs is mediated, in large part, by induction of apoptosis within the target cells [31,32]. Caspase 3 is one of the key enzymes in the CTL-induced apoptosis pathway. This assay involves labeling the target cells with cell tracker dye then culturing them with CTLs to activate apoptosis, staining the cells with antibody-recognized cleaved caspase 3, and analyzing by flow cytometry [30,33]. Some practical limitations of this flow cytometry-based assay are that the number of harvested immune cells is often insufficient for flow cytometry. Immune cells must be stimulated and cultured to obtain sufficient numbers, which can distort the cells’ phenotype and function [34]. Also, because it is a measure of caspase 3 activation, this assay is not suitable for the caspase-independent pathway of target cell killing by CTLs [35].

### 2.5. Cytokine Production Assays

Cytokines are hormone-like proteins that enable immune cells to communicate. They play an integral role in initiating, perpetuating, and subsequently controlling the immune response. Cells can communicate with one another by direct contact or through secretion of soluble mediators. Direct cell-to-cell contact regulates the immune function of adjacent cells by a variety of mechanisms, including membrane-bound cytokines. In contrast, soluble mediators permit cells to exert influence at a distant site within a tissue, or even affect cells in other organs via the peripheral circulation [36]. Measuring cytokine production provides functional information about the immune system, and perhaps indirect evidence of immune-cell activation. Cytokines secreted by cells of the immune system can alter the behavior and properties of immune or other cells. At a site of inflammation, sets of cytokines interact with immune cells, and their combined effect is often more important than the function of one isolated component. The frequencies of cytokine-producing cells can be measured by ELISPOT or by flow cytometric analysis of intracellular cytokines.

ELISPOT assays employ either a monoclonal or polyclonal capture antibody that is coated aseptically onto a polyvinylidene fluoride (PVDF)-backed microplate. Cytokines (or other cell products of interest) secreted by activated cells are captured locally by the coated antibody on the high-surface-area PVDF membrane. A second biotinylated antibody reactive to a distinct epitope of the target cytokine is added, and thus is employed to detect the captured cytokine. After washing to remove any unbound biotinylated antibody, the detected cytokines are visualized using an avidin-HRP and a precipitating substrate. The colored end product (usually a blackish blue spot) typically represents an individual cytokine-producing cell. The spots can be counted and sized manually or with an automated reader. The ELISPOT assay determines antigen-specific T-cell activation through IFN-γ production of individual cells in response to a tumor associated antigen (TAA)-specific APC*,* which correlates with CTLs’ ability to lyse cells bearing such TAAs in vivo [37,38,39]*.* The majority of ELISPOT assays are conducted to measure IFN-γ-secreting cells, but antibody pairs have been developed to measure other cytokines such as TNF-α, IL-4, and IL-5. A significant advantage of ELISPOT is that limits of detection are low, ranging from 1:300.000 to 1:100.000 [39]. Although this can be an effective way of assessing immune response associated with clinical benefit, it has several limitations, including significant variability from laboratory to laboratory and from plate reader to plate reader [40]. The ELISPOT assay, when routinely executed by experienced personnel, was found to be highly reproducible, with an interassay coefficient of variation of 15%. Its sensitivity was found to be 1/100,000 cells [41]. ELISPOT assay is not useful with whole tumor cell vaccines or nonspecific immunotherapies, such as cytokines or antibodies, since these therapies are not TAA-specific [42,43,44]. Attempts to standardize or automate ELISPOT assays are currently ongoing [45].

Intracellular cytokine staining by flow cytometry is a powerful technique that allows the analysis of individual cells in a mixed population. Intracellular cytokine assays are rapid and quantitative, and provide information on the functional role of CTLs in various conditions. However they have a limit of detection of >1 antigen-specific T cell per 10,000 peripheral blood mononuclear cells [46]. The procedure relies on the stimulation of T cells in the presence of an inhibitor of protein transport, in order to retain the cytokines inside the cell. Cells are first stimulated with antigen, followed by staining with antibodies specific for extracellular epitopes, such as CD4 and/or CD8. The frequency of cells that produce a particular cytokine is measured using fluorescent antibodies. Intracellular cytokine staining can be used as an immune monitoring tool to measure the immune response to known antigens, or to identify and/or validate novel T-cell epitopes [47]. Nonspecific background staining is the major drawback of cytokine flow cytometry.

Many other methods have been employed to evaluate cytokine production in response to immunotherapy. Enzyme-linked immunosorbent assay (ELISA) was introduced in the 1970s. The antibody attached to the bottom of a well provides both antigen capture and immune specificity, while another antibody linked to an enzyme provides detection and signal amplification. This approach enables accurate and sensitive detection of the antigen, the cytokine of interest [48]. Because of these features, ELISA has been considered the standard method of cytokine measurement. At the same time, several weaknesses have been recognized. ELISA’s performance is largely dependent on antibody quality, kit manufacturer, and operator skills and experience [49]. In addition, ELISA permits the measurement of only one cytokine at a time. Difficulties also exist in comparing two cytokine levels measured by two different ELISAs, each under somewhat different conditions [48]. In summary, cytokine detection by ELISA is highly variable among different patients, and overall sensitivity is low.

In contrast, real-time polymerase chain reaction (RT-PCR) analysis, which is highly sensitive and reproducible, can evaluate cytokine production by measuring mRNA. As a method of single-cell detection, RT-PCR is unsuitable for screening large numbers of cells, but has the unique ability to measure multiple mRNA species in individual activated T cells [50]. A significant concern, however, is the extreme sensitivity of this assay, raising the possibility of measuring low-level, biologically insignificant transcriptional “noise.” Another drawback of this technique is that mRNA analysis necessitates destruction of immune cells, which prevents determination of T-cell specificity [46].

### 2.6. Immunosuppression Assays

In advanced tumors, there is disequilibrium between the tumor and immune recognition, allowing tumors to escape immune recognition and proliferate uncontrollably [51]. The tumor microenvironment contains not only CD8^+^ T cells and memory effector cells, but also immunosuppressive cells such as FoxP3^+^ Tregs [52], myeloid-derived suppressor cells [53], tumor-associated macrophages [54], and cancer-associated fibroblasts [55]. These immunosuppressive cells inhibit antitumor immunity by secreting cytokines such as IL-10 and TGF-β, which further attenuates the effect of CTLs [56,57]. Tumor cells and other cells in the tumor microenvironment secrete cytokines such as IL-6 [57], indolamine-2,3-dioxygenase [58], vascular endothelial growth factor [59], programmed death-1 ligand [60], and soluble Fas ligand [61], which hamper antitumor immunity and promote tumor growth. A significant factor in the inability to mount optimal immune responses to an immune-based therapy is immunosuppression by these cells and their cytokines. Thus, for a vaccine, an immune stimulating therapy, evaluation of pre-treatment status of immune-suppressive cells, such as Tregs, would be informative, and may provide predictive information to vaccine’s efficacy.

There are several ways to assess immunosuppressive cells, the most common being quantification and functional analysis of Tregs. Quantification analyses are usually done with flow cytometry to detect cells that are CD4^+^CD25^high^FoxP3^+^, the most accepted marker of Tregs [62]. One of the limitations of this approach is the small population of FoxP3^–^ Tregs [62]. Flow cytometry analysis of peripheral blood obtained from prostate cancer patients and healthy donors revealed that the percentage of CD4^+^CD25^high^FoxP3^+^ Tregs was not significantly different between the 2 groups [63]. Tregs’ functionality was analyzed by determining their ability to suppress the proliferation of CD4^+^CD25^−^ T cells [63]. This assay used ^3^H thymidine incorporation, similar to the T-cell proliferation assay described above. The results showed that Tregs from patients with prostate cancer had a significantly greater suppressive function than Tregs from healthy donors (*p* < 0.05). In addition, while patients treated with PSA-targeting vaccine showed no significant change in the number of Tregs, Treg functionality decreased post-vaccination, with a trend in the correlation between survival benefit and decreased Treg suppressive function post- *vs*. pre-vaccination [64,65].

### 2.7. Humoral Response

There are many methods for detecting serum antibodies against a particular antigen, including, but not limited to, ELISA and immunoprecipitation. Most of these methods use the binding capacity of antibodies against an antigen to detect and quantify their concentration. Additionally, antigen array technology provides a high-throughput approach to identifying many different antigens in one assay [66]. When a particular antibody is identified retrospectively using array technology, it must then be validated prospectively as an endpoint in a clinical trial. Findings from the clinical trials of sipuleucel-T and PSA-TRICOM suggest that monitoring serum antibody levels may be a useful indicator for patients receiving vaccines.

The phase III trial of sipuleucel-T (IMPACT) identified antibody titers >400 to PA2024, in 100/151 patients (66.2%) in the treatment arm compared with 2/70 (2.9%) in the placebo group [1]. Antibody titers of ≥400 against PAP at any time after baseline were seen in 43/151 patients (28.5%) in the treatment arm compared with 1/70 (1.4%) in the placebo arm. In a prespecified analysis, patients with antibody titers (as measured by ELISA) of >400 against PA2024 or PAP had improved survival compared to those with antibody titers of ≤400 (*p* < 0.001 and *p* = 0.08, respectively, by log-rank test). In a recently reported, pooled immune analysis of three randomized controlled trials of sipuleucel-T (IMPACT, D9901, and D9902A) [28], overall survival correlated significantly with the development of at least one post-baseline peripheral immune response to PA2024 or PAP (HR 0.47, *p* = 0.003), PA2024 (HR 0.46, *p* = 0.002), and PAP (HR 0.53, *p* = 0.019). The strongest correlation with overall survival was seen with the development of antibody responses to PA2024 at any time point (HR 0.42, *p* < 0.001).

## 3. Promising Techniques That May Suggest Potential Benefit from Immune-Based Therapies

### 3.1. Immune Infiltrates in Tumors

The immune assays described above are designed to detect immune responses in peripheral blood. Regardless of their statistical significance, these assays do not provide direct evidence for antitumor activity of activated immune cells within tumors. Examination of the tumor and its microenvironment following treatment with a therapeutic cancer vaccine is thus highly desired. In a trial of neoadjuvant sipuleucel-T (NCT00715104), patients scheduled for radical prostatectomy were treated with 3 infusions of sipuleucel-T prior to surgery. Prostatectomy specimens were examined to assess the immune response within prostate tissue in comparison to the core biopsy sample. The preliminary report suggested that neoadjuvant sipuleucel-T was associated with increased intratumoral frequency of total T cells, T helper cells, CTLs, and Tregs, specifically at the interface between benign and malignant tissue. These data suggest that sipuleucel-T can modulate the presence of lymphocytes in prostate cancer tissue *in vivo* [67]. These findings may provide information of immune infiltrates within the tumors. Identification of these immune cells would be informative in elucidating the mechanisms of action [68].

Tumor-infiltrating lymphocytes have been shown to be an independent indicator of improved prognosis in several tumor types, including ovarian cancer [69], endometrial carcinoma [70], melanoma [71], colorectal cancer [72,73], and pleural mesothelioma [74]. These reports suggest that subpopulations of T cells, such as CD8^+^ T cells and effector memory T cells that drive type 1 immunity, appear to correlate with improved clinical outcome. By contrast, infiltration of Tregs is associated with poorer clinical outcome in many cancer types, such as breast cancer [75], non-small cell lung cancer [76], gastric cancer [77] and renal cell carcinoma [78]. In a study in breast cancer patients, high numbers of infiltrating FoxP3^+^ Tregs was associated with a higher risk of relapse after 5 years [75].

The major limitation of this approach is the technical difficulty of accessing the tumor and getting sufficient tissue for various immune cell analyses. The process may require harvesting, culturing, stimulation, and purification of the collected immune cells, which may distort the phenotype of the original cells. In a study of intraprostatic administration of PSA-targeted vaccine in 21 patients with locally recurrent prostate cancer, patients received a priming vaccine subcutaneously, followed by 3 intraprostatic booster vaccinations at 4-week intervals [79]. Patients underwent optional biopsies pre- and post-vaccination. A paired t-test of 13 pre- and post-vaccination biopsies showed significant increases in immune infiltrates within tumors after vaccination. CD4^+^ cells increased from 1.3 to 13.1/high power field (hpf). CD8^+^ cells rose from 6.4 to 14/hpf. The sample size was too small to make any clinical correlation.

### 3.2. Tumor Growth Rates

A recent analysis of prostate cancer trials suggest that the potential delayed effect seen with therapeutic cancer vaccines may ultimately impact the tumor growth rate. That is to say that the immune response generated may not shrink existing tumor but may ultimately slow the growth of cancer, which could have a more long-lasting impact. This hypothesis is based on a recent analysis of 5 prostate cancer trials conducted at the National Cancer Institute [80], all of which were in metastatic castration-resistant prostate cancer. Four of the trials employed standard therapeutics; the fifth used the therapeutic cancer vaccine PSA-TRICOM. Using a previously established mathematical equation that employs PSA to determine tumor growth rate and ultimately predict mortality, it was determined that the 4 standard therapy trials had predictable outcomes [81]. Tumor growth rate was significantly changed while patients were on therapy; however, when the therapy was discontinued, the growth rate resumed at its pretreatment rate and mortality was predictable using the established equation. The findings with vaccine were somewhat different. While patients were on study (a median of 3 months), no change was observed in tumor growth rate, which is consistent with the lack of change observed in time to progression [64,80]. But there was a change in survival that was not predicted by the off-treatment growth rate. In the patients treated with vaccine, survival was well beyond what would have been predicted using the off-study growth rate and the same mathematical equation. This result suggests a biological interplay between the immune system and tumor that, over time, can alter growth rate in a manner that is different from standard therapeutics, resulting in an effect that is sustained beyond the period of vaccine treatment.

Although this hypothesis needs to be confirmed in prospective, randomized trials, it does provide a potential explanation for the quandary of how immunotherapies can improve survival without changing short-term progression. If prospective trials do in fact demonstrate that measurable growth rates are altered by immune therapies, it may be possible to use disease-specific parameters such as PSA in prostate cancer, M-spike in multiple myeloma, and tumor size in renal cell cancer as surrogates for response to treatment [81]. This would allow practitioners to determine which patients are showing altered tumor growth rates, and therefore are responding to therapy, distinguishing them from patients requiring other therapeutic interventions.

## 4. Conclusions

One of the greatest limitations to developing an intermediate biomarker of response for immunotherapy has been the lack of clinical efficacy. With the emergence of sipuleucel-T, ipilimumab, and other immunotherapies in late stages of clinical testing, there will likely be greater opportunities to compare clinical outcomes and potential biomarkers. Given the number of available biomarker assays, many of which are undergoing modernization to improve accuracy while limiting variability, it would seem possible that some biomarkers will be key to predicting response to immunotherapy. It is equally possible, however, that the complexity of the immune response is such that it cannot be simply evaluated by one or two biomarkers. Given the heterogeneity of tumors, the ultimate benefit of immunotherapy may be the variability of its effect on the immune system from patient to patient, which may result in improved long-term outcomes. In that case, approaches such as intratumoral immune infiltrates and altered growth rates may have high potential. It is also possible that if less toxic immunotherapies, such as therapeutic cancer vaccines, work best in minimal disease states, their ultimate utility may be as part of adjuvant or neoadjuvant therapy, which may mitigate the need for biomarkers [82]. While the ultimate role of biomarkers in immunotherapy remains unclear, immunotherapies themselves have earned a vital role, along with chemotherapy, hormonal therapy, and targeted therapies, in the treatment of cancer.
